# Fulminant hepatic failure (FHF) due to acute hepatitis C

**DOI:** 10.12669/pjms.314.7618

**Published:** 2015

**Authors:** Bilal Bin Younis, Rozina Arshad, Saima Khurhsid, Junaid Masood, Farhan Nazir, Maham Tahira

**Affiliations:** 1 Bilal Bin Younis, Sakina Institute of Diabetes and Endocrine Research, Shamalar Medical & Dental College Hospital, Lahore, Pakistan; 2 Rozina Arshad, Sakina Institute of Diabetes and Endocrine Research, Shamalar Medical & Dental College Hospital, Lahore, Pakistan; 3 Saima Khurhsid, Sakina Institute of Diabetes and Endocrine Research, Shamalar Medical & Dental College Hospital, Lahore, Pakistan; 4 Junaid Masood, Sakina Institute of Diabetes and Endocrine Research, Shamalar Medical & Dental College Hospital, Lahore, Pakistan; 5 Farhan Nazir, Sakina Institute of Diabetes and Endocrine Research, Shamalar Medical & Dental College Hospital, Lahore, Pakistan; 6 Maham Tahira, Sakina Institute of Diabetes and Endocrine Research, Shamalar Medical & Dental College Hospital, Lahore, Pakistan

**Keywords:** Hepatitis, Fulminant hepatic failure, HCV

## Abstract

Acute hepatitis C (HCV) infection has been identified as an important cause of fulminant hepatic failure (FHF), characterized by rapid deterioration of liver function from massive hepatic necrosis leading to encephalopathy and multi-organ failure. We admitted a female patient at Shalamar Hospital with jaundice, fever, encephalopathy and coagulopathy of short duration with no history of any comorbidity. Her hepatitis viral screen revealed positive anti HCV. Her viral loads were also high. A diagnosis of FHF due to acute HCV infection was made. Patient was treated conservatively and improved gradually. In summary, acute HCV can cause FHF and should be ruled out in patients with FHF of unknown cause in an endemic country for HCV like Pakistan.

## INTRODUCTION

Fulminant hepatic failure (FHF) is defined as the rapid acute liver injury with rapid deterioration of liver functions and hepatic encephalopathy in a patient without apparent, prior liver disease. There are several definitions of FHF based on the time of onset of symptoms like jaundice, fever and development of encephalopathy. Although hyper acute, fulminant and subfulminant hepatic failure may differ in their presentation, like the development of cerebral edema, renal failure or portal hypertension, in clinical practice these sub definitions are not traditionally accepted.^1^

Viral hepatitis is the most frequent cause of FHF. Around 40–60% of patients with FHF, thought to be due to some viral infection, were found to have negative serological markers for hepatitis A virus (HAV) and hepatitis B virus (HBV), being classified as non-A non-B (NANB) hepatitis which are now called Hepatitis C Virus–Associated Fulminant Hepatic Failure.^2^

Hepatitis C virus (HCV) has been identified as a major cause of NANB Acute hepatitis. HCV infection has been identified as an important cause of FHF, characterized by rapid deterioration of liver function from massive hepatic necrosis leading to encephalopathy and multiple organ failure with a high mortality rate of 65 to 92%.^3^,^4^

## CASE REPORT

An 80 years old woman was admitted to female medical ward at Shalamar Hospital Lahore with history of yellow discoloration of sclera for 15 days and altered state of consciousness for 10 days and history of melena for one day. She had no history of other comorbidities like diabetes, hypertension or chronic liver disease. Clinically she was vitally stable having deep jaundice and flapping tremors. Liver was palpable one finger below the costal margin tender having soft consistency. At the time of admission her lab investigations including complete blood urea, creatinine and serum electrolytes were normal LFTs showed total billurubin 6.9mg/dl, ALT 1560 U/L, AST 1398U/L, alkaline phosphatase 540 U/L, albumin 2.5 g/dl, PT prolonged 3 sec, USG shows hepatomegaly with total liver span of 13.5cm with fatty change. Her viral hepatitis screen including Anti HAV, Anti HEV and HBsAg were negative, these tests were performed by using electrochemiluminescence technology. Her Anti HCV was positive with cut off value of 1.00 and patient value of 10.17. Her HCV RNA by PCR was detected with viral load of 6.1 x 10^5^ IU/ml. She developed progressive encephalopathy and coagulopathy. Her peak serum bilirubin was 15mg/dl. Due to deranged coagulation profile liver biopsy was relatively contraindicated. She was treated conservatively, and patient gradually improved and discharged from hospital.

The diagnosis of fulminant hepatitis due to acute hepatitis C was based on the assessment of clinical, virologic measures. None of the medications that were administered to the patient were known to be hepatotoxic. There was least possibility of drug induced hepatitis as patient first presented in our clinic with no previous visits to any doctor or hospital stay in the previous six months. She was not taking any homeopathic, Unani medicines and also not taking regular allopathic medications. None of the serum samples had detectable levels of hepatitis B surface antigen, IgM antibodies against hepatitis A or hepatitis E. LFTs done in this patient strongly supported acute hepatic insult. The autoimmune hepatitis is very rare in this age group and no other signs of autoimmune disease were found. We should have excluded the possibility of co-infection with hepatitis G virus (HGV) but we had no facility to perform Anti HGV in Pakistan besides the prevalence of acute hepatitis G in still unknown in Pakistan. Based on these observations diagnosis of FHF due to hepatitis C was made.

## DISCUSSION

Pakistan is an endemic area for HCV and HBV. A study from Pakistan reported the prevalence of 3.17% of hepatitis B and 13% of hepatitis C among the high risk people.^5^ According to another study HBsAg and Anti-HCV incidence was 2.6% and 5.3% in normal healthy adults, 13.0% and 10.3% in high risk groups, and 25.7% and 54% in patients with chronic liver disease respectively. This study tells the high endeminicity of viral infections in Pakistan where hepatitis B and C potentially account for a serious burden of the disease.^6^

In developing countries like Pakistan viral hepatitis is the most common cause of acute liver failure and among these viral infections hepatitis A is more common^7^ whereas hepatitis B and C are more associated with chronic hepatitis. In United States, drugs and toxins are thought to be the most common cause of fulminant hepatic failure and viral infections. Hepatitis B is probably the most common viral cause of FHF and the incidence may be underestimated, since many cases do not undergo routine serology.^8^

The role of hepatitis C in fulminant hepatic failure is still controversial in a study by Chu CM et al.^9^ 40 to 60% of patients with FHF thought to be due to viral infections were found to be having negative serology for viral markers being classified as non A non B virus (NANB). Recently, Hepatitis C is recognized as a major cause of community acquired NANB. Perhaps the most important correlation of hepatitis C, FHF is the presence of concurrent chronic hepatitis B infection. This study shows that acute HCV infection superimposed upon chronic HBV infection significantly increases the risk of FHF. However, in our patient other viral markers including hepatitis B were negative. In another study, 32.6% patients considered to have acute hepatitis C were found to have HCV RNA positive and out of these, 34% were positive for anti-HCV. None of the patients with acute hepatitis A or B were HCV RNA positive, while HCV RNA was detected in 70 of 128 patients with acute NANB hepatitis.^9^ Serum HCV RNA were found in 40 to 60 percent in Japan and Taiwan, but in only 2 percent in Western countries which may reflect geographic differences in the epidemiology of HCV infection or the pathogenicity of the prevalent viral strains.

Further investigations are required to elucidate the mechanisms by which HCV infection results in fulminant hepatic failure. However, co-infection or super infection of HCV and other hepatitis viruses may play an important part in the development of this fatal disease. In our patient we could not rule out the possibility of co-infection with HGV as we have no facility to detect anti-HGV.

In summary, HCV can cause fulminant hepatic failure. The disease is characterized by continuous viral replication. The detection of serum HCV RNA by PCR is the earliest and most valuable marker for the diagnosis of fulminant hepatitis C.
